# Dr. Young's Address to Certain Reviewers

**Published:** 1814-01

**Authors:** Thomas Young

**Affiliations:** Worthing


*For the Medical and Physical Journal.*

A LETTER *to the* EDITORS *of the* EDINBURGH MEDICAL *and* SURGICAL JOURNAL.

GENTLEMEN,

NOTWITHSTANDING that I have been so unfortunate as to incur your displeasure, by involuntarily becoming the object of some very high encomiums which have been bestowed on me, perhaps too lavishly, in a popular review, while, as you have clearly proved from my own words, the nature of the subject itself rendered perfection unattainable, I still depend so much on your regard for your own general character, which I am by no means disposed to depreciate, as to believe that you will take the earliest opportunity to correct a very important error, into which you have fallen, in asserting, that I have recommended to a student of physic to attend an hospital "in the second year *only*" of his medical studies. Now, in the 18th page of my Essay you will find these words: "As the spring advances, he must become a pupil of an hospital, which must continue to be his principal and *daily* object at *every subsequent* period, *while* practical lectures should be attended with diligence;" and these lectures are enumerated in the following page, among the pursuits of the third year. The public must be the judges between us; and how you will be able to exculpate yourselves before them for so unaccountable a misrepresentation, which you have expanded into a whole page of argument, I am utterly at a loss to foresee. You have either censured what you have not read, or what you have read and forgotten; for I cannot believe you both mischievous and foolish enough to advance such an accusation, at the moment that you were fully aware of its falsehood.

Whether or no you were in the same happy predicament of inattention or oblivion when you asserted that I spoke "*lightly* of the clinical lectures of Edinburgh," I will not pretend to determine: perhaps you will solemnly maintain that to mention in express terms their "*excellence*," and their "*acknowledged superiority*," (p. 15) is to speak lightly of them; but this is more a matter of opinion than the former, and therefore I shall not expect you to retract it. It is, however, an absolute mis-statement to say that I have considered the affections comprehended under the term PNEUSIS as one DISEASE; for I have most carefully remarked (p. 28) that I have intentionally united *different* diseases, as species constituting the same *genus*, according to their natural analogies, and to the most convenient mode of treating of them:

nor nor have you been more correct in insinuating that the gout is to be found in two places, without *any* reference from one to the other; for in fact there are references in *both* the passages, in which I have thought it necessary to mention respectively the different forms of this very anomalous disease.

The charges which you have grounded on these supposed offences, do not very materially affect the general merits of the work; nor should I think its credit much diminished by admitting the truth of all else that you have advanced against it, especially if you succeeded in convincing your readers that "it ought to be in the library of every medical scholar to whom the work of Ploucquet is inaccessible," and that its value is not altogether "superseded even by that stupendous work;" but having been induced to point out the most palpable of your mistakes, I shall briefly examine such others of your objections as appear most to require an answer.

You observe, without entering into the merits of the "detached essays," that they are "excrescences in such a work." Is a rose then an excrescence upon a myrtle, because they happen to be planted in the same bed? You seem unwilling to allow that any work in *English* on the study of physic can be required; and mention at the same time the Bibliotheca of Haller and the Index of Ploucquet, in order to deprive me of every excuse for publishing any thing not absolutely perfect. I can only observe that I have not copied a *single word* from either of those works; and how either of them is to be employed "as a guide to students," I am at a loss to understand; but on the mere circumstance of the language being English rather than Latin, I never meant to found any particular claim to indulgence. You do not appear to be aware that a methodical arrangement of every department of my subject is one of my professed objects, and the correct accentuation of names, contemptible as you may deem it, another: both these objects require in all cases a systematic enumeration, at least, of the whole nomenclature of the respective sciences, even when there are few or no works to be referred to each article; and in some such cases, you have very candidly produced the empty shelves of the library as affording fair specimens of its contents. The repetitions concerning "exotic medicine" exist only in your imagination. With respect to the alphabetical order, for which you express a decided preference, it can have no advantage over the worst system furnished with a good index; and if a general or scientific view is to be taken of any part of medical knowledge, it appears to me so evident as to require no proof, that it can only be obtained by means of a systematic systematic arrangement. You object to my conversion of generic into trivial names; but, in fact, if we wish to find a disease from its name only, we must look for it in an index, and then it is of no importance whether the name is generic or trivial; if from its nature and symptoms, we must consult the definitions, and the alternative remains equally indifferent. When you say that I have made a classification of names, and not of diseases, I am utterly incapable of comprehending your meaning, nor can I easily believe that you had yourselves any distinct idea of what your words were intended to express. You assert that my genera are founded on forced analogies; but in proof of this assertion you adduce no argument, and scarcely even an example, except in the case of the arrangement of the Aphtha of Cullen as a typhus, in which, though with some hesitation, I have followed Dr. Crichton: on this occasion you very concisely and I suppose satisfactorily answer my *note of interrogation* by a *note of admiration*, and there the illustration ends. That "the mildest and most terrible of diseases are the children of one family," is certainly not more unnatural than that an equal diversity should occur in the same individual disease, that is, in the same species; for instance, no fever can be slighter than some cases of scarlatina, and none can be more inevitably fatal than others. You disapprove my having "melted down Dr. Willan's orders into genera;" but I had the pleasure to find, in conversation with that respectable author before his death, that he himself considered it as a very proper adaptation of his partial method to a more extensive system, which he had the goodness to say he considered as extremely neat, and altogether unexceptionable: indeed it is impossible to read his account of what he calls species, without perceiving that a great number of them are mere varieties of the same disease. Almost all the instances of apparent repetition that you have pointed out, relate to cutaneous diseases, in which, as I have expressly observed, "I have thought it better to follow Dr. Willan's arrangement with very little alteration, than to attempt to introduce definitions and subdivisions more strictly methodical:" sometimes, too, a solitary and accidental affection of a small part of the surface of the body may, very properly, be considered as belonging to a distinct class from that which comprehends affections of the same kind which are more extensive and habitual.

To your accusation of having made my "generalisations in the closet," I must certainly plead guilty. I have been accustomed to observe the progress of single cases at "the bed-side," and in the theatre; but where I have wished to consider consider the relations of a hundred different cases of a hundred different diseases, I should have thought it perfectly absurd to sacrifice the convenience and tranquillity of the closet, for the fanciful advantage of being at the bed-side of one of the ten thousand cases, at the moment that I labored to obtain a perception of the general relations of the whole.

The term "Therapeutics," which you seem to prefer to "Acology," had already been employed in a different sense, and is usually understood as relating rather to the general principles of the practice of physic, than to the separate consideration of the operation of particular remedies. I allow that I have not divided the sedatives, commonly so called, according to the nature of their immediate action, but have been contented with referring back to the whole catalogue from the exhaurients: I really found it so difficult to satisfy myself how they ought to be divided, that I despaired of untying the knot, and was afraid of doing harm by attempting to cut it, as it was perhaps my duty to have done. I have not indeed professed myself by any means satisfied with the arrangement of this division of my work, which, however, I am happy to find you have thought more deserving of your indulgence than the rest; for, however I may think the value of your commendations diminished by the want of a sounder spirit of criticism, and a greater power of discrimination than you appear to me to have displayed in your censures, I do not profess to be altogether indifferent to your good opinion.

I am, Gentlemen,

*Worthing*, Your very obedient Servant,

*Oct.* 21, 1813. THOMAS YOUNG.

